# The 2014 Ebola outbreak: ethical use of unregistered interventions

**DOI:** 10.2471/BLT.14.145789

**Published:** 2014-09-01

**Authors:** Ruediger Krech, Marie-Paule Kieny

**Affiliations:** a World Health Organization, avenue Appia 20, 1211 Geneva 27, Switzerland.

The large number of cases and wide geographical spread distinguish the current 2014 outbreak of Ebola virus disease in west Africa from all known earlier outbreaks.^1^ In the past, outbreaks of this disease have been stopped by identifying all cases, tracing all contacts and making sure that those caring for patients use correct protective gear at all times. However, the success of such methods depends on the presence of: (i) functional health systems; (ii) health workers who are trained, paid, willing to be deployed and adequately protected in a dangerous work environment; (iii) experts in public health with the skills needed to manage the tracing of people and monitor the evolution of the disease effectively; and (iv) people with solid skills in social engagement and development who are available to work with at-risk communities.^2^ Such systems and individuals were largely absent from the area where the current outbreak of Ebola virus disease is believed to have begun – a border area between three countries that all have fragile health systems and that are emerging from the traumas of civil war.

Encouragingly, research efforts over the past decade have led to the development, for the first time, of a range of potential treatments and vaccines that could support efforts to control Ebola virus disease. However, although some of these interventions have proven effective in animal models, none has completed clinical testing in humans – a step that is indispensable for the registration of any medical intervention as proven and safe. Why have there been no clinical trials, given that we have known the Ebola virus for 40 years? Why is there no effective registered vaccine or treatment available? At the onset of the current Ebola outbreak – despite some resources provided by the governments of Canada and the United States of America – substantial financial investment was still needed to evaluate and develop several interventions for the control and treatment of Ebola virus disease. Until now – as seen with several other neglected diseases – this disease has received little attention because it was affecting mostly poor people in poor countries.

The above shortcomings aggravate an ethical dilemma. If the treatments for Ebola virus disease that are currently under development could save lives – as the results of animal studies indicate – should they not be used immediately, since far too many people have already died? On the other hand, if there is a possibility that a treatment might cause substantial adverse effects in humans that have not been seen in animal testing, should it not be withheld?^3^

On 11 August 2014, the World Health Organization (WHO) convened a consultation to consider and assess the ethical implications of the potential use of unregistered interventions, such as drugs, vaccines and passive immunotherapy, in the current Ebola outbreak. The results of this consultation have been widely discussed in the media.^4^

In summary, the consultation’s panel of experts advised WHO that, in the particular circumstances of the current outbreak – and provided certain conditions are met – it would be ethical to offer unproven interventions – with as yet unknown efficacy and adverse effects – for the potential treatment or prevention of Ebola virus disease. One of the conditions that need to be met is that ethical principles must guide the provision of such interventions. For example, there must be transparency about all aspects of care, informed consent, freedom of choice, confidentiality, respect for the person, preservation of dignity, and involvement of the community.

To understand the safety and efficacy of these interventions, the panel of experts advised that – when and if any of the unregistered interventions is used to treat patients – there is a moral obligation to collect and share all of the data generated, including data arising from any treatment provided for compassionate use – i.e. the use of an unregistered drug outside of a clinical trial.^5^

What can we learn from this crisis? Robust health systems are key for controlling disease outbreaks. Let us make sure that development efforts are designed to strengthen health systems. Well trained and motivated health workers are indispensable. They should be paid and receive the support they need to carry out their duties. And, finally, increasing investment into research and development for the treatment, control and prevention of diseases that currently mostly affect poor people and poor countries should be a key priority for policy-makers worldwide. Let us not forget these lessons when the current Ebola outbreak no longer appears on the front pages of our newspapers.
